# Intraosseous catheter confirmation with central venous color flow and doppler waveform, a randomized single-blinded trial

**DOI:** 10.1186/s13054-026-05979-x

**Published:** 2026-04-07

**Authors:** Eric S. Kretz, Ian Hudson, Titus Chu, Ryan P. Dumas, Jennifer Achay, Scotty Bolleter, Brayden Salas, Michelle Huerta, Emily Epley, David Wampler

**Affiliations:** 1 Department of Emergency Medicine, Brooke Army Medical Center, San Antonio, TX USA; 2 UT Health San Antonio, Emergency Health Sciences, San Antonio, TX USA; 3 Centre for Health Sciences, Bulverde, TX USA; 4 Department of Emergency Medicine, Wright State University, Dayton, OH USA; 5 Division of Trauma and Acute Care Surgery, Baylor College of Medicine, Houston, TX USA

**Keywords:** Intraosseous, POCUS, Color flow, Doppler wave form, Resuscitation, Shock

## Abstract

**Background:**

Obtaining intraosseous (IO) access is an established procedure within the critical care setting. Mechanisms for routinely confirming IO placement and sustained IO efficacy lack objectivity, fail to incorporate modern technology, and lack protocol advancement that improves patient treatment, safety, and provider confidence. In this study, we investigate the novel use of color and doppler point-of-care ultrasound (POCUS), in combination with an IO fluid bolus, as a potential means to confirm IO placement.

**Methods:**

*Design*: randomized single-blinded comparison using doppler and color flow to confirm proximal central venous flow during IO infusion. *Study model*: fresh, never frozen, unembalmed, and consented cadavers. *Standard arm*: intentionally correct IO placement randomized across the proximal humerus, distal femur, and proximal tibia. *Comparison arm*: intentionally incorrect IO placement. *Protocol*: Following access confirmation, an IO fluid bolus was initiated. An operator, blinded to the correct versus the intentionally incorrect IO placement, applied POCUS to the central vein proximal to the established IO catheter. POCUS, in concert with a fluid bolus, was then used to assess for color flow and the generation of a doppler waveform at the associated proximal central vein. The operator additionally applied POCUS to assess the IO insertion site and extremity for the presence of extravasation.

**Results:**

The unblinded operators identified proximal venous color flow and doppler waveform in 100% of intentionally placed IOs, while the blinded POCUS operator identified them in 86% and 88% of cases with correctly placed IOs. 7% intentionally misplaced IOs generated doppler waveform and color flow, ultimately determined on post-procedure dissection to be secondary to an inadvertent cannulation of a deep peripheral vein adjacent to the bone. On average, correctly placed tibial IOs required more volume to produce color and doppler flow than humoral IOs.

**Conclusion:**

The ability to accurately confirm and reassess IO catheter placement and patency is of vital importance throughout the entire critical care continuum. Awareness of procedural POCUS specific to the use of color flow and doppler waveform to confirm IO placement may provide additional assurance that an IO catheter has been successfully placed, remains in position, and is functioning safely.

## Background

Obtaining emergent intraosseous (IO) access is an established procedure in critical care. Furthermore, in a large multicenter trial, IO was found to be quicker and more successful than peripheral intravenous (IV) or central venous access [1]. Traditional mechanisms for confirming successful IO placement remain historically static with rudimentary, best practice reliance on tactile feedback, catheter stability, and the ability to aspirate blood. Once an IO is established, clinicians are instructed to visually observe the insertion site for evidence of soft tissue swelling and the arbitrary flow of fluids. Unsuccessful, misplaced, or dislodged IO catheters can lead to clinically significant complications if unrecognized [2–6].

Procedural point-of-care ultrasound (POCUS) is now broadly available across most training and clinical settings, including prehospital and hospital-based clinicians as an established tool that improves procedural skill and confirmation. The current standard of care includes POCUS to obtain and occasionally confirm successful peripheral and central venous access [13, 15]. Prior research has looked at the role of POCUS in the assessment of IO access related to the intramedullary space, with widely variable sensitivity and specificity, in both swine and small cadaver studies [7–12].

A large cadaver study successfully demonstrated an increase in proximal vein circumference directly related to an IO fluid bolus [14]. Here, we sought to evaluate the role of color and doppler waveform ultrasound analysis on proximal central veins during IO fluid bolus administration. We hypothesized that a successfully placed IO, with an associated fluid bolus, will dynamically generate proximal central vein doppler waveform and color flow.

## Methods

### Ethics

The UT Health San Antonio Institutional Review Board reviewed and waived this study as non-human subject research (IRB ID STUDY00001178). Specimens were requested and approved through the Texas State Funeral Commission and the University of Texas Southwestern Willed Body Program.

### Clinical trial number

Not applicable.

### Study design

We conducted a cadaveric-based, blinded, control trial that compared successfully placed IO catheters to intentionally misplaced IO catheters.

### Cadaver model and randomization

**Fig. 1:**
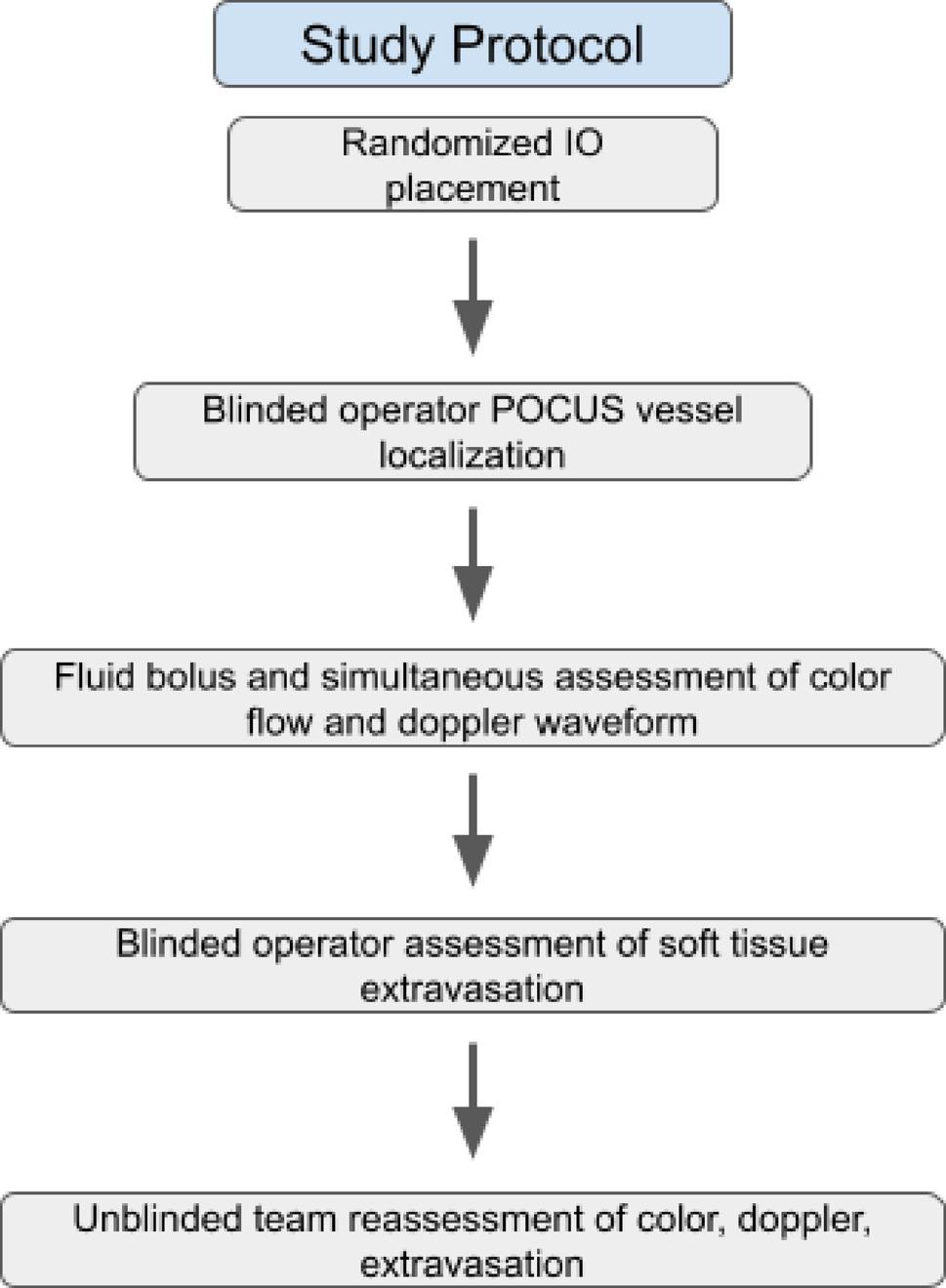
Study Flow

Twelve adult, fresh, recently deceased, non-frozen, unembalmed, consented cadavers of any age, weight, and gender were sequentially enrolled in this study. Cadavers were excluded if IO access would be contraindicated in situ; exclusions included: significant orthopedic-related surgeries, recent fractures, or vascular compromise. Anatomical sites (proximal humerus, distal femur, or proximal tibia; 1:1:1) were randomized, and a 5:1 ratio of successful and misplaced IO catheters were also randomized. A 5:1 ratio was selected to approximate published IO access success rates expected by an emergency physician in a clinical setting. After randomization, IO needle sets were placed using a 15 gauge 45 mm needle set (EZ-IO Teleflex Morrisville, NC, USA). Successful IO catheter placement was confirmed utilizing the traditional means of (1) tactile appreciation upon entry into the intramedullary space, (2) catheter stability in the bone, and (3) the ability to aspirate blood from the catheter. Intentional misplacement of needle sets was performed by the use of a 15 gauge 45 mm IO needle-set (EZ-IO TeleFlex Morrisville, NC, USA), placed entirely through the bone and into adjacent soft tissues. Confirmation of failed placement included (1) tactile appreciation of an incorrectly performed insertion, (2) catheter stability in the bone, and (3) the inability to aspirate blood (Fig. 1).

### Blinding

A POCUS experienced board-eligible emergency physician was blinded to both. (A) successful and (B) intentionally misplaced randomization.

### POCUS assessment

**Fig. 2:**
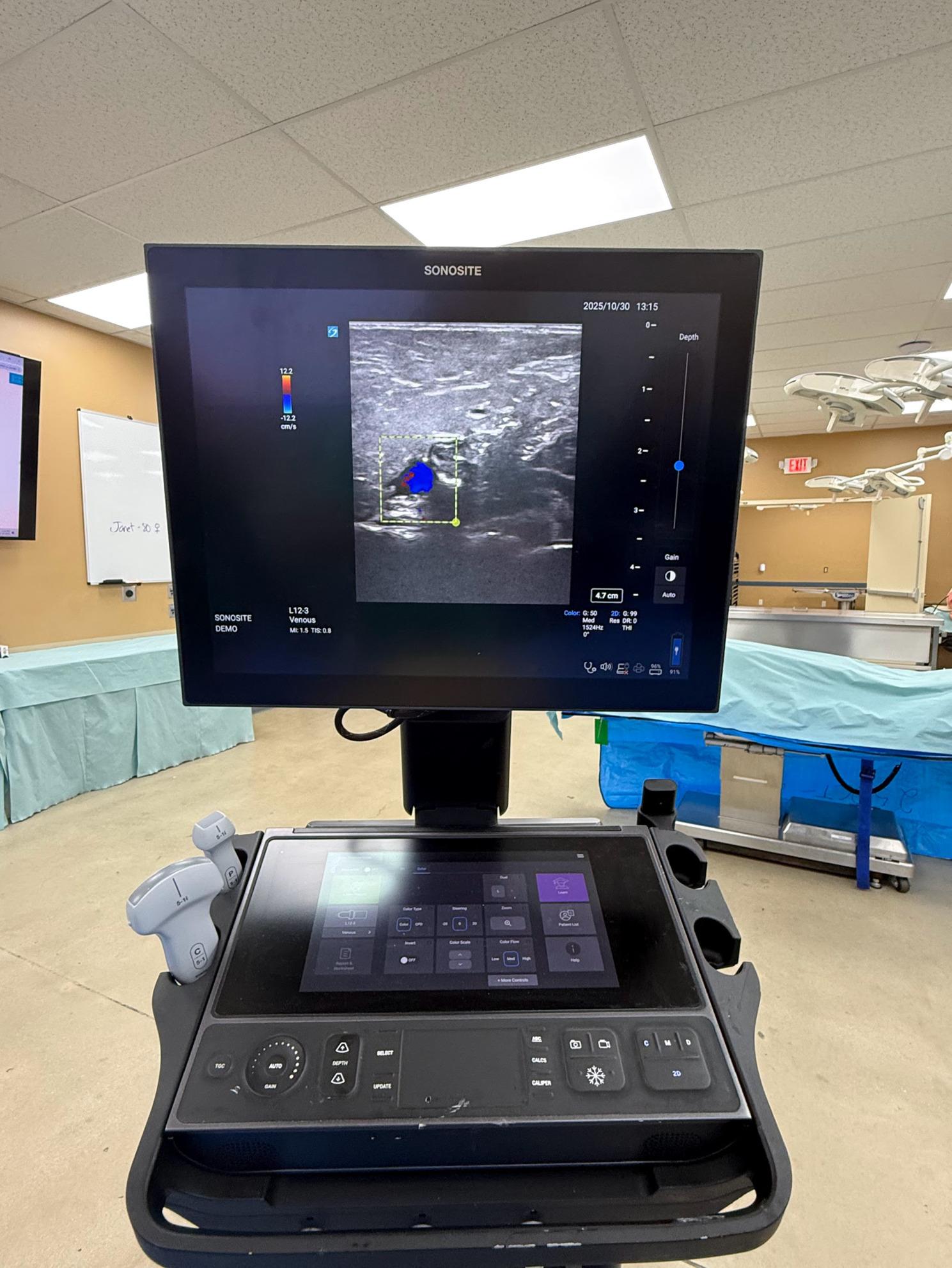
Color flow during a fluid bolus through a correctly placed IO

**Fig. 3:**
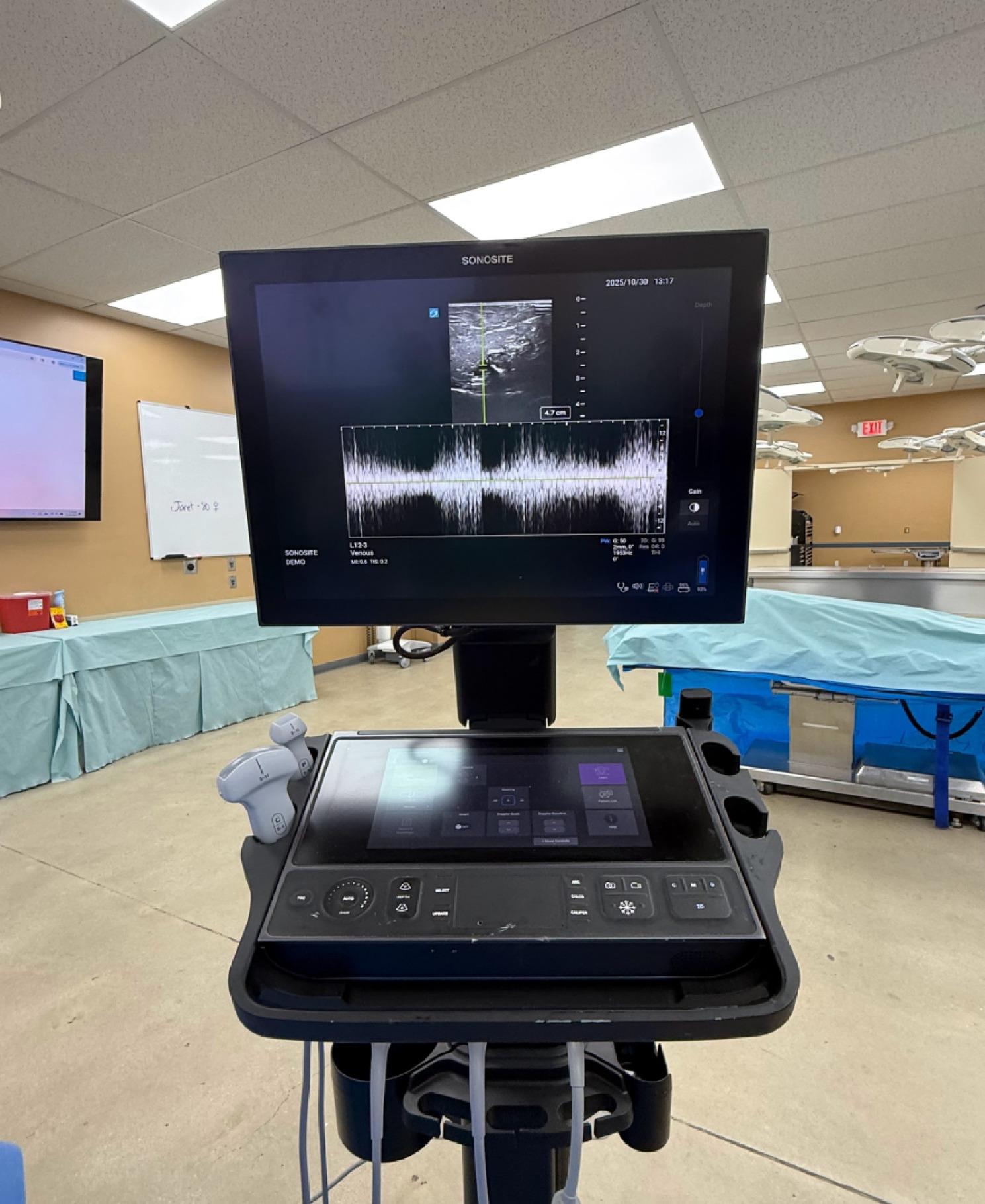
Doppler flow during a fluid bolus through a correctly placed IO

The blinded operator trained in the use of ultrasound (Sonosite LX FUJIFILM Bothell, WA, USA) with simultaneous split-screen doppler waveform and color flow identified the central vein proximal to the IO site (subclavian vein for the humerus, and femoral vein for the distal femur and proximal tibia IOs). Flow was then initiated using normal saline bolus through the IO catheter with a manual syringe pump (LifeFlow Durham, NC, USA). The operator recorded if visualization of doppler and/or color flow occurred until 60 ml of flow was reached Figs. 2, 3.

Upon completion of each iteration, placement was again confirmed by study staff in absence of the blinded operator. Placement was confirmed as described above for pretest confirmation.

### Statistical analysis

Descriptive statistics were used to perform demographic analysis. Inference was done using chi square for categorical data. Paired two-sided t-test was used for continuous data. IBS SPSS and Microsoft Excel were used to analyze the obtained data statistically. By convention, alpha was set to 0.05.

## Results

In 12 cadavers, 24 IO catheters at each anatomical site (right and left), for a total of 72 IO catheters placed Table 1. There were 58 IO catheters correctly placed (19 in the proximal tibia, 20 in the proximal humerus, 19 in the distal femur). There were 14 IO catheters intentionally misplaced (5 in the proximal tibia, 4 in the proximal humerus, 5 in the distal femur) All correctly established IO catheters were tactilely inserted, demonstrated stability in position, and unblinded investigators were able to aspirate blood. All misplaced IO catheters were tactilely inserted (noting two distinct “drops” or “gives” confirming placement through and through the bone), were stable in the bone, and unblinded investigators were not able to aspirate blood.

**Table 1:** Cadaver Demographics

		Count	Mean	95.0% lower	95.0% upper
				CL for mean	CL for mean
Sex	Female	42			
	Male	30			
Age			83	82	84
Heigh (in)			66.7	65.5	67.9
Weight (lbs)			154	143	166

### Overall and comparison

The pooled sensitivity and specificity of color doppler during IO bolus across all sites were 87.9% (CI:0.7955–0.9631) and 92.9% (CI:0.7937–1.0635). The pooled sensitivity and specificity of the doppler waveform during IO bolus across all sites were 86.2% (CI:77.3–95.1) and 92.9% (CI: 79.4-1.0635.4.0635). On unblinded investigator reassessment, 100% of successfully placed IOs generated color flow and a doppler waveform. Generation of a doppler waveform during IO bolus had a positive predictive value of 98% and a negative predictive value of 62%; while generation of color doppler had a positive predictive value of 98% and a negative predictive value of 65%.

A one-way ANOVA showed a significant difference in volume to detect color flow in correctly placed IO catheters among the three groups (F(2,3.300), *p*= 0.045). Post hoc Tukey test indicates that the mean volume required to identify color flow was significantly higher in the tibia (M = 27.50mL, 95% CI: 20.90–34.10) than in the humerus (M = 17.89mL, 95% CI: 13.47–22.32), while the volumes needed to identify color flow were not significantly different between the humerus-femur or tibia-femur.

A one-way ANOVA showed a significant difference in mean volume required to detect a doppler waveform among the three groups (F(2,4.048), *p*= 0.024). Post hoc Tukey test indicates that the volume required to identify color flow was significantly higher in the tibia (M = 32.00mL, 95% CI: 25.32–38.68) than in the humerus (M = 21.58mL, 95% CI: 16.69–26.47), while the volumes needed to identify color flow were not significantly different between the humerus-femur or tibia-femur.

### Color flow, doppler waveform, extravasation

#### Distal femur IO catheters

The blinded POCUS operator identified both color flow and generation of a doppler waveform in 16 (84%) of the 19 correctly placed distal femur IO catheters. 0 of 5 (0%) of the intentionally misplaced distal femur IO catheters produced either color flow or a doppler waveform. Soft tissue extravasation was observed in 4 of 19 (21%) correctly placed IO catheters and 3 of 5 (60%) misplaced IO catheters.

#### Proximal humerus IO catheters

The blinded POCUS operator identified both color flow and generation of a doppler waveform in 19 (95%) of the 20 correctly placed proximal humerus IO catheters. Of the misplaced IO catheters 0 of 4 (0%) of the proximal humerus placements produced either color flow or a doppler waveform. Soft tissue extravasation was reported in 2 of 20 (10%) correctly placed IOs, and 4 of 4 (100%) misplaced IOs.

#### Proximal tibia IO catheters

The blinded POCUS operator identified color flow and generation of a doppler waveform in 15 and 16 (79% and 84%) of the 19 successfully placed proximal tibia IO catheters. Of the misplaced proximal humerus IO catheters, 1* of 5 (20%) produced both color flow and a doppler waveform. Soft tissue extravasation was reported in 3 of 19 (16%) correctly placed IOcatheters and 3 of 5 (60%) intentionally misplaced IO catheters.

### Bolus volume

The blinded POCUS operator was able to identify color flow and doppler waveform in successfully placed IO catheters at variable volumes depending on the IO insertion site.

The blinded operator identified color flow in correctly placed humeral IOs with an average bolus volume of 17.89mL (95% CI: 13.47–22.32) and doppler flow with an average bolus volume of 21.58mL (95% CI: 16.69–26.47). Paired two-sided t-test shows a significant difference between average volume required to detect color 17.89mL (95% CI: 13.47–22.32) and doppler flow 21.58mL (95% CI: 16.69–26.47) (t(18) = 2.11, *p* = 0.49).

The blinded operator identified color flow in correctly placed femoral IOs with an average bolus volume of 25.00mL (95% CI: 17.98–32.02) and doppler flow with an average bolus volume of 31.88mL (95% CI: 23.80–39.95.80.95). Paired two-sided t-test shows a significant difference between average volume required to detect color 25.00mL (95% CI:17.98–32.02) and doppler flow 31.88mL (95% CI: 23.80–39.95.80.95) (t(16) = 3.801, *p* = 0.002).

The blinded operator identified color flow in correctly placed tibial IOs with an average bolus volume of 27.50mL (95% CI: 20.90–34.10) and doppler flow with an average bolus volume of 32.00mL (95% CI: 25.32–38.68). Paired two-sided t-test shows a significant difference between average volume required to detect color 27.50mL (95% CI: 20.90–34.10) and doppler flow 32.00mL (95% CI: 25.32–38.68) (t(14) = 3.674, *p* = 0.003.

These findings suggest that, in addition to tactile placement, catheter stability, and the aspiration of blood, more than a 10mL but less than 40mL bolus would be needed to confirm IO catheter patency.

## Discussion

This study demonstrated that a fluid bolus administered through a properly placed intraosseous (IO) catheter produces both Doppler waveform and color flow within proximal central veins. Verification of proximal flow is clinically important, as critically ill patients often require fluid or blood resuscitation and vasoactive medications during multiple transitions of care (e.g., EMS to ED, ED to ICU, floor to ICU, ED to surgery, surgery to ICU). IO access provides a rapid and reliable method for emergent vascular access and has been shown to serve as an effective bridge to more definitive access [1, 14]. In trauma populations, IO placement has been reported to be faster and more successful than alternative vascular access methods [1]. Findings from this study suggest that, alongside traditional techniques for confirming IO placement, point-of-care ultrasound (POCUS) assessment of proximal venous color flow and Doppler waveform generation during an IO-related fluid bolus are sensitive indicators of correct catheter placement and maintenance. Successful use of POCUS in this context requires operators to possess a solid understanding of anatomy and IO-specific ultrasound skills.

*Saab et al.*. successfully demonstrated that an IO catheter related fluid bolus increases the circumference of proximal central and peripheral veins, leading to our hypothesis that color and doppler waveform would be generated from a similar fluid bolus. The results of this study demonstrate a similar relationship between IO infusion volume and proximal venous flow that was reported by Saab, et al. The generation of color flow and doppler waveform in proximal central veins should give providers reassurance regarding IO catheter placement and patency. With dissection, we identified one case of an intentionally misplaced IO catheter, which generated color wave flow and doppler waveform during a fluid bolus, resulted from an inadvertent cannulation of the posterior tibial vein. Given that the peripheral veins drain into the same central vein, this particular misplaced IO catheter subsequently produced a similar set of POCUS images. Further, while doppler waveform and color flow are largely objective measures of venous flow, unblinded, experienced investigators were able to appreciate the impact that unintentional artifacts, predominantly operator movement, had in creating an appearance similar to dynamic flow in false positive cases. While cadaveric vascular hemodynamics do not identically match that of living patients with native blood flow, given that a manual calf squeeze generates substantial venous change on central venous POCUS, the authors suspect that a fluid bolus in a living patient with spontaneous circulation would produce similar results to those found in this study [16].

Findings from this study suggest that, alongside traditional techniques for confirming IO placement, point-of-care ultrasound (POCUS) assessment of proximal venous color flow and Doppler waveform generation during an IO-related fluid bolus are sensitive indicators of correct catheter placement and maintenance. These results have particular relevance for protracted emergency room holds, prolonged field care, and battlefield medicine, where throughput problems or evacuation delays and limited resources demand reliable, durable vascular access. In such environments, the ability to confirm IO function using POCUS could enhance provider confidence, reduce complications, and support sustained resuscitative efforts until definitive treatment is achieved. Further, these results are directly related to patient safety at the junction of prehospital and hospital-based care and intra-hospital unit-to-unit transfers.

It is important to note that unblinded co-investigators reassessed IO catheter placement after the blinded investigator’s initial assessment. Of the IO catheters correctly placed and reassessed, 100% (58/72) generated a doppler waveform, color flow and no sign of extravasation. Of the intentionally misplaced IO catheters 7% (1/14) generated color flow and doppler waveform *but also caused* extravasation. This suggests that the sensitivity and specificity of POCUS confirmation may be dependent on operator POCUS-specific IO training and experience.

### Limitations

Catheter adjacent or superficial tissue extravasation and deep tissue or compartment extravasation are established concerns during the use of an IO catheter [2, 6, 9, 17]. The visual identification of catheter adjacent extravasation for a misplaced or dislodged IO catheter is an important finding, but singularly incomplete. Thorough and dynamic POCUS assessment of associated compartments is equally important, yet can be misleading secondary to gravity-dependent pooling or a slow infusion rate. This study was confounded by several cadavers presenting with atypical 3rd spacing of fluid into soft tissue compartments when compared to living patients, which likely had some impact on the evaluation of fluid extravasation. Given this understanding, the investigators employed a combined catheter adjacent or superficial assessment, a careful assessment of compartment tissues, as well as doppler waveform and color flow. Additionally, the blinded POCUS operator in this study was a board-eligible emergency physician proceeding without inter-operator reliability or POCUS artificial intelligence (AI) assistance. Given that this study was designed to utilize a single blinded POCUS operator, and current technology lacks IO associated POCUS AI, assessment collaboration and/or assistance for IO catheter patency was not evaluated.

## Conclusion

The ability to gain, sustain, and readily reconfirm IO access is critical for both resuscitation and the patient’s safe progress through various critical transitions of care. Traditional methods for IO catheter confirmations and its continued suitability for use may fall short of patient need and modern technology. As such, the traditional IO access confirmation may be further enhanced with the addition of POCUS color and doppler waveform assessment of central veins, proximal to the established IO, while in association with a small fluid bolus. This combined assessment may improve patient safety, provider confidence., and serve as a more specific and reliable indicator of successful and sustained IO catheter placement.

## Data Availability

The dataset from this study is available from the corresponding author upon reasonable request.

## Abbreviations

AI: Artificial intelligence
ED: Emergency department
EMS: Emergency medical services
ICU: Intensive care unit
IO: intraosseous
IV: Intravenous
POCUS: Point of care ultrasound
